# An Edge-Enhanced Network for Polyp Segmentation

**DOI:** 10.3390/bioengineering11100959

**Published:** 2024-09-25

**Authors:** Yao Tong, Ziqi Chen, Zuojian Zhou, Yun Hu, Xin Li, Xuebin Qiao

**Affiliations:** 1 School of Artificial Intelligence and Information Technology, Nanjing University of Chinese Medicine, Nanjing 210023, China; yaotong@njucm.edu.cn (Y.T.); zhouzj@njucm.edu.cn (Z.Z.); huy@njucm.edu.cn (Y.H.); 2 Jiangsu Province Engineering Research Center of TCM Intelligence Health Service, Nanjing University of Chinese Medicine, Nanjing 210023, China; 3 Vanke School of Public Health, Tsinghua University, Beijing 100084, China; chenzq21@mails.tsinghua.edu.cn; 4 College of Computer Science and Software Engineering, Hohai University, Nanjing 211100, China; li-xin@hhu.edu.cn; 5 School of Elderly Care Services and Management, Nanjing University of Chinese Medicine, Nanjing 210023, China

**Keywords:** polyp segmentation, convolutional neural network, edge enhancement, attention mechanism

## Abstract

Colorectal cancer remains a leading cause of cancer-related deaths worldwide, with early detection and removal of polyps being critical in preventing disease progression. Automated polyp segmentation, particularly in colonoscopy images, is a challenging task due to the variability in polyp appearance and the low contrast between polyps and surrounding tissues. In this work, we propose an edge-enhanced network (EENet) designed to address these challenges by integrating two novel modules: the covariance edge-enhanced attention (CEEA) and cross-scale edge enhancement (CSEE) modules. The CEEA module leverages covariance-based attention to enhance boundary detection, while the CSEE module bridges multi-scale features to preserve fine-grained edge details. To further improve the accuracy of polyp segmentation, we introduce a hybrid loss function that combines cross-entropy loss with edge-aware loss. Extensive experiments show that the EENet achieves a Dice score of 0.9208 and an IoU of 0.8664 on the Kvasir-SEG dataset, surpassing state-of-the-art models such as Polyp-PVT and PraNet. Furthermore, it records a Dice score of 0.9316 and an IoU of 0.8817 on the CVC-ClinicDB dataset, demonstrating its strong potential for clinical application in polyp segmentation. Ablation studies further validate the contribution of the CEEA and CSEE modules.

## 1. Introduction

Colorectal cancer (CRC) is among the top three most prevalent cancers globally and ranks second in cancer-related mortality rates [1,2,3,4]. Fortunately, research has demonstrated that early screening and endoscopic polypectomy are crucial in reducing CRC incidence [5,6]. A critical aspect of these procedures is the accurate localization of polyps, which aids in their removal. However, this task is tedious and time-consuming in clinical practice, particularly during early screenings, which may generate over 10,000 images per patient [7,8,9]. With advances in computer-aided diagnostic technologies [10,11,12], developing an accurate and real-time automated polyp segmentation framework offers a promising solution to assist clinicians in distinguishing polyp from non-polyp regions.

Polyp segmentation poses distinct challenges compared to other medical segmentation tasks, primarily due to the variability in polyp appearance (e.g., differences in size, color, and texture) [13] and the minimal contrast between polyps and surrounding tissues [14,15,16]. To address the multi-scale nature of polyps, recent studies have introduced a series of multi-scale feature aggregation methods [17] to effectively merge high-level features without imposing excessive computational demands [13,18,19,20,21]. Meanwhile, efforts to resolve the low contrast problem have focused on capturing discriminative regions using spatial attention mechanisms or boundary constraints [22,23,24]. For example, LDNet [25] introduced a lesion-aware cross-attention mechanism to enhance feature contrast between polyp and non-polyp areas.

Recent advancements in polyp segmentation, particularly transformer-based models such as Polyp-PVT [26], MGCBFormer [27], MIA-Net [28], and CAFE-Net [29], have demonstrated the efficacy of transformers in handling long-range dependencies and multi-scale feature extraction. However, these models often face challenges in boundary refinement, particularly for smaller or ambiguous polyps. In response to these challenges, we propose a novel architecture that explicitly enhances edge features and integrates cross-scale edge information, offering improved segmentation accuracy, especially in boundary precision.

While current methods have shown success, they struggle to match the precision of expert physicians, particularly when it comes to accurately identifying polyp boundaries—a complex task even for experienced clinicians. This is of significant clinical concern, as polyps often appear on mucosal surfaces, and improved boundary segmentation is essential for minimizing damage to surrounding tissues during polyp removal. Two primary issues contribute to the coarse segmentation of polyp boundaries: (i) inadequate boundary awareness and (ii) insufficient contrast between the boundary and surrounding regions. First, the limited amount of annotated polyp data leads to poor boundary awareness in models, as the diversity of shapes and sizes in the training set is restricted. While Guo et al. [30] attempted to address this with a confidence-guided label mixup strategy, their approach reduced contrast between polyp and non-polyp regions and introduced inaccurate supervision in uncertain areas, which could hinder model training. Second, the subtle contrast in colonoscopy images makes boundary features less distinguishable, often resulting in wavy or inaccurate predictions. Although recent methods have employed spatial attention mechanisms to enhance polyp features, these strategies have primarily focused on the polyp’s interior, leaving the boundary contrast issue largely unresolved.

To address these challenges, we propose an edge-enhanced network (EENet) for polyp segmentation. To be specific, we design a covariance edge-enhanced attention (CEEA) module to capture edge context by attentive covariance analysis. Moreover, a cross-scale edge enhancement (CSEE) module is proposed to bridge the gap of edge context between encoded and decoded feature maps. By flexibly embedding CEEA and CSEE into the encoder–decoder framework, sufficient edge details are preserved and utilized to produce a high-certainty polyp boundary. The main contributions are as follows:

1. We propose CEEA, which integrates edge-awareness with covariance-based attention. The CEEA module introduces a learnable Canny kernel [31] for adaptive edge detection, allowing the network to focus on fine-grained boundaries and structures crucial for accurate segmentation. By leveraging a covariance matrix between the feature map and edge-enhanced feature, the module captures both spatial and channel dependencies, enhancing the attention mechanism’s ability to focus on relevant regions.
2. We introduce CSEE to fuse cross-scale features with edge-enhanced attention. The module uses a shared learnable Canny kernel to extract edge information at different scales, allowing the model to capture fine-grained boundary details across resolutions. By computing a cross-scale attention map, the CSEE module ensures that features from both encoder and decoder paths are aligned, enhancing the representation of critical structures such as object edges.
3. We design a hybrid loss function that incorporates edge and cross-entropy losses with a handcrafted hyperparameter. By appending the CEEA of each encoder stage and deploying CSEE between the encoder and decoder, the proposed EENet enables the improvement of boundary accuracy while maintaining multi-scale consistency, leading to better segmentation performance.
4. Through extensive experiments on two benchmark datasets [32,33], EENet demonstrates superior performance over state-of-the-art models. Our results are further validated by an ablation study that highlights the advantages of CEEA over convolution layer and conventional self-attention models. Furthermore, we test the effects of CSEE in EENet.

The paper is structured as follows. Section 2 provides an overview of related works in polyp segmentation. Section 3 introduces the EENet with its sub-modules. Section 4 presents the comparisons and ablation studies. Section 5 draws the conclusion of our work and points out the future directions.

## 2. Related Works

### 2.1. Traditional Methods for Polyp Segmentation

Early approaches to polyp segmentation primarily employed traditional image processing and machine learning techniques [34,35,36]. These methods often relied on handcrafted features and predefined rules, such as edge detection, threshold-based segmentation, and morphological operations. For example, Xia et al. [37] utilized a method that first identified a preliminary region of interest (pROI) using a modified Hough transform. After removing the background, a two-step process was implemented: a relaxation technique to segment homogeneous regions, followed by a refinement step to merge unnecessary segments based on color differences in the CIE color space. In another approach, Wang et al. [38] proposed a computer-aided detection (CAD) system for identifying colorectal polyps by analyzing both local and global geometric features of the colon wall. This system employed texture and morphological information to quickly detect suspicious regions, using edge detection and an elliptical polyp model to quantitatively evaluate the identified areas. To reduce false positives, the method incorporated both texture and morphological features. Similarly, Jerebko et al. [39] introduced a method for polyp detection that utilized symmetric curvature patterns to differentiate polyps from other intestinal structures. By extracting symmetry-based curvature features from candidate regions, this method aimed to enhance detection sensitivity. However, traditional approaches struggled to address the wide variability in polyp shape, size, and texture, resulting in limited effectiveness when applied to real-world scenarios [40,41,42,43].

### 2.2. Deep Learning Methods for Polyp Segmentation

Convolutional neural networks (CNNs) have greatly improved polyp segmentation by offering more flexible and reliable methods for analyzing medical images [44,45,46]. One of the most influential models, U-Net [47], features a fully convolutional architecture that integrates local and global features via its encoder–decoder framework. The use of skip connections between corresponding layers helps retain spatial details, making U-Net particularly effective for medical segmentation tasks that demand both detailed and broader contextual understanding [24,48]. Building on U-Net, several models have emerged to further improve segmentation performance. For example, UNet++ [15] employs nested skip pathways to reduce the semantic gap between the encoder and decoder, enhancing the model’s capacity to capture fine-grained features. Its multi-resolution feature fusion strategy allows UNet++ to process complex medical images more effectively, leading to improved segmentation accuracy and robustness. Another significant extension, ResUNet [49], integrates residual blocks from ResNet into the U-Net architecture. These residual blocks mitigate the gradient vanishing issue during deep network training, thus improving the network’s ability to extract complex features. The ResUNet has demonstrated particular effectiveness in handling medical images with intricate backgrounds and subtle structural differences. These U-Net-based architectures, by incorporating innovations such as skip connections, multi-scale feature fusion, and residual learning, have significantly advanced polyp segmentation performance. Despite these improvements, researchers continue to explore novel architectures and techniques to push the limits of segmentation accuracy and robustness in clinical settings. Specifically, unique challenges, such as the considerable variation in polyp appearance and the low contrast between polyps and surrounding tissues, persist, necessitating specialized approaches. To address the diversity in polyp shape and size, Wang et al. [50] proposed a multi-scale context-guided framework that captures both global and local features, allowing the model to handle objects of varying scales. Similarly, ThresholdNet [51] introduced a confidence-guided label mixup technique that augments the training dataset, enhancing the model’s generalization capability across different polyp shapes and sizes.

Low contrast between polyp boundaries and surrounding areas presents another challenge. PraNet [21] tackled this issue by utilizing a reverse attention mechanism to progressively enhance the discriminative polyp regions. Similarly, LDNet [25] designed a lesion-aware cross-attention mechanism to improve contrast between the polyp boundary and its surrounding tissues, aiding in more accurate boundary detection.

Recent advancements in medical image segmentation have been significantly influenced by the introduction of Vision Transformers (ViTs) [52] and Pyramid Vision Transformers (PVTs) [53], which offer enhanced capabilities in modeling long-range dependencies and multi-scale context aggregation [54,55]. ViTs, as introduced by Dosovitskiy et al. [52], utilize a self-attention mechanism to effectively capture global relationships in images by processing patches, thus overcoming the limitations of CNNs in capturing global context. Similarly, PVTs, proposed by Wang et al., integrate the strengths of CNNs and transformers by employing a hierarchical structure that captures both local details and global context at multiple scales.

These transformer-based models have achieved state-of-the-art performance in various segmentation tasks [17,56], including medical image segmentation. For example, TransUnet [57] combines the global context modeling ability of transformers with the high-resolution features of CNNs in the decoder. In the study by Dong et al. [26], the Polyp-PVT model demonstrated superior performance in polyp segmentation, achieving an average Dice score of 0.917, significantly outperforming previous methods like PraNet and U-Net. Similarly, MGCBFormer [27] utilizes multi-scale grid priors and boundary-aware mechanisms to further refine segmentation precision, addressing both boundary detection and contextual feature extraction. Meanwhile, MIA-Net [28], developed by Liu et al., combines both transformers and convolutional layers to enhance feature learning, offering a balanced approach that captures both local and global features, thus improving segmentation accuracy. Lastly, the CAFE-Net [29] model proposed by Zhao et al. introduces cross-attention and feature exploration techniques to enhance polyp segmentation, particularly by focusing on hard-to-detect regions. These models, while advancing the state of the art, also highlight the ongoing challenge of balancing high segmentation accuracy with computational efficiency, a critical factor for real-time clinical applications.

Despite these advancements, many approaches continue to struggle with accurately segmenting polyp boundaries, especially in challenging clinical scenarios. These limitations underscore the need for further research to improve boundary segmentation performance in polyp detection tasks.

## 3. Method

### 3.1. Covariance Analysis

In this subsection, we introduce the covariance analysis [58,59,60], which is central to the functioning of the proposed CEEA module in our EENet framework. The motivation for using covariance analysis stems from the need to capture spatial and channel dependencies within the feature maps, especially in tasks like polyp segmentation where boundary precision and fine-grained spatial relationships are critical.

Covariance analysis helps capture the relationships between channels in the input and edge-enhanced feature maps by quantifying how two feature channels vary together. This is crucial for highlighting areas in the feature map important for accurate polyp boundary detection, where low contrast with surrounding tissues often leads to segmentation errors.

By incorporating covariance into the attention mechanism, the network can learn to focus on both the channel dependencies and the spatial regions that contribute the most to the segmentation task. This process ensures that the network assigns higher weights to feature channels and regions that highlight the polyp’s boundaries, which are typically difficult to distinguish.

The covariance analysis plays a pivotal role in enhancing polyp segmentation by improving the model’s ability to capture both spatial and channel dependencies. By leveraging covariance matrices, the network can more effectively by carrying out the following:

1. Focusing on boundary regions that are difficult to distinguish due to low contrast;
2. Assigning attention weights that prioritize channels and spatial regions relevant to polyp boundaries;
3. Ensuring that both fine-grained details and global contextual information are integrated into the segmentation process, leading to higher accuracy and improved boundary delineation.

Therefore, this method is particularly important in the context of medical image segmentation, where small, subtle differences between polyps and surrounding tissues can significantly impact clinical outcomes.

### 3.2. Overview of the Proposed EENet

**Figure 1:**
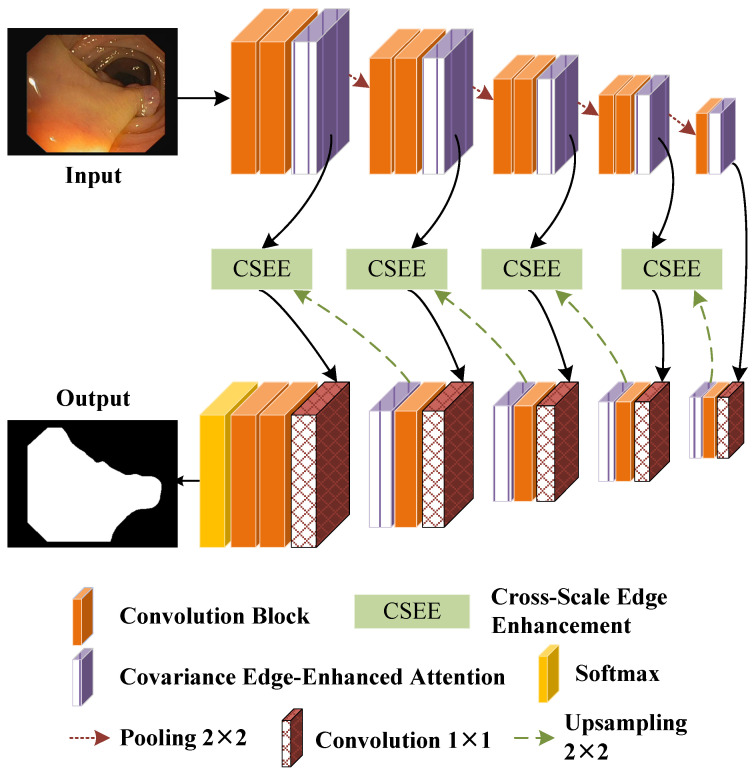
The framework of the EENet.

**Figure 2:**
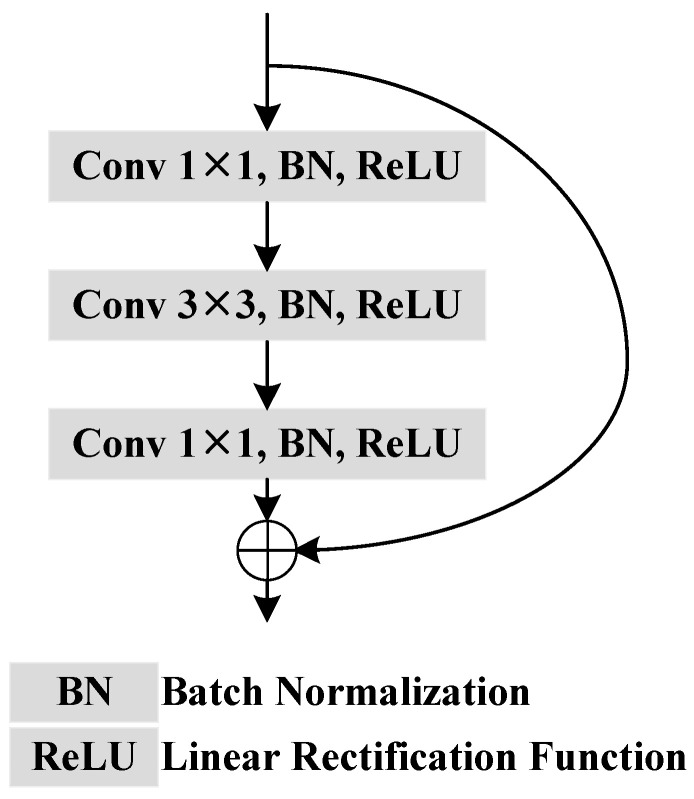
Pipeline of the CB.

As shown in Figure 1, we introduce the architecture of the proposed EENet designed for accurate polyp segmentation. The EENet framework is structured around an encoder–decoder architecture, where the integration of edge enhancement at multiple scales is key to improving boundary precision. The core components of the EENet include the CEEA module and the CSEE module, which work synergistically to enhance edge features and preserve fine boundary details during segmentation. The EENet architecture consists of several convolutional blocks, each incorporating batch normalization and ReLU activation functions (see in Figure 2).

CEEA enhances feature extraction by capturing both spatial and channel dependencies through covariance analysis. This allows the network to attend to crucial regions, particularly around polyp boundaries, which are often difficult to distinguish due to low contrast with surrounding tissues. On the other hand, to bridge the gap between the encoder and decoder stages, the CSEE module is introduced. The CSEE is responsible for fusing multi-scale edge features from different levels of the network, ensuring that fine-grained boundary information is preserved throughout the segmentation process.

Finally, the Softmax function is applied to generate pixel-wise semantic predictions. Furthermore, to train the EENet effectively, we propose a hybrid loss function that combines cross-entropy loss with edge-aware loss.

While transformer-based architectures like Polyp-PVT and MGCBFormer effectively capture global context, they often require auxiliary post-processing or additional mechanisms to handle boundary refinement. Our proposed CEEA module introduces a novel approach to edge extraction during feature encoding, which directly improves boundary precision. Additionally, the CSEE module dynamically integrates edge information across multiple scales, addressing a key limitation in models like MIA-Net and CAFE-Net, where cross-scale edge integration is not explicitly modeled. This results in better performance, particularly in difficult polyp boundary delineation tasks.

### 3.3. Pipeline of the Proposed CEEA

**Figure 3:**
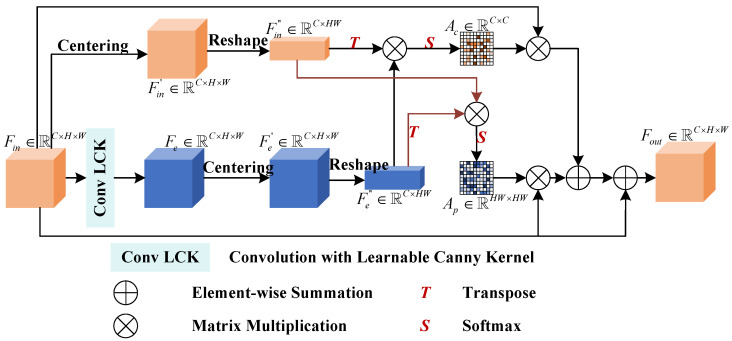
Pipeline of CEEA.

The CEEA module is a crucial part of the EENet, designed to capture fine-grained boundary details and spatial-channel relationships using covariance-based attention. The CEEA integrates a learnable Canny kernel to adaptively detect edges in the input feature maps, enhancing the edge-related features essential for accurate segmentation. In this section, we provide a detailed explanation of the pipeline of the proposed CEEA module, illustrated in Figure 3.

Given the input feature map, $F_{in}\in R^{C\times H\times W}$, where *C*, *H*, and *W* represent the number of channels, height, and width, respectively, a convolution layer with a learnable Canny kernel is implemented to extract edge features from $F_{in}$, termed as follows:

(1) $$F_{e}={\mathrm{Conv}}_{LCK}\left(F_{in}\right),$$

where $F_{e}\in R^{C\times H\times W}$ is the edge-preserved feature map. This operation adapts the standard Canny edge detection algorithm by learning the optimal edge detection parameters directly from the data, thereby making it more flexible in handling diverse polyp structures.

After edge feature extraction, the module applies a covariance-based attention mechanism to capture both spatial and channel dependencies. The covariance matrix is computed between the edge-preserved feature map, $F_{e}$, and the input feature map, $F_{in}$. First, we compute the mean for each channel of both the input feature map, $F_{in}^{\prime \prime }$, and the edge-enhanced feature map, $F_{e}^{\prime \prime }$:

(2) $${\bar{F}}_{in,c}=\frac{1}{HW}\sum_{i=1}^{HW}F_{in,c,i}$$

(3) $${\bar{F}}_{e,c}=\frac{1}{HW}\sum_{i=1}^{HW}F_{e,c,i}$$

Subsequently, we subtract the mean values from the corresponding feature maps to center them around zero:

(4) $$F_{in}^{\prime }=F_{in}-{\bar{F}}_{in,c},$$

(5) $$F_{e}^{\prime }=F_{e}-{\bar{F}}_{e,c},$$

where $F_{in}^{\prime }$ and $F_{e}^{\prime }$ are the centered feature maps. Afterwards, we reshape these two feature maps to $F_{in}^{\prime \prime }\in R^{C\times HW}$ and $F_{e}^{\prime \prime }\in R^{C\times HW}$, respectively. Then, the covariance matrix is computed as follows:

(6) $$\mathrm{Cov}{\left(F_{in},F_{e}\right)}_{c_{1},c_{2}}=\frac{1}{HW}\sum_{i=1}^{HW}F_{in,c_{1},i}^{\prime \prime }\cdot F_{e,c_{2},i}^{\prime \prime },$$

where $\mathrm{Cov}\left(F_{in},F_{e}\right)\in R^{C\times C}$ is the covariance matrix, where each element represents the covariance between a channel from $F_{in}$ and a channel from $F_{e}$, $F_{in,c_{1},i}^{\prime \prime }$ denotes the centered input feature at channel $c_{1}$ and spatial location *i*, and $F_{e,c_{2},i}^{\prime \prime }$ represents the centered edge-preserved feature at channel $c_{2}$ and spatial location *i*. Thus, the attention map of channel-wise covariance can be expressed as follows:

(7) $$A_{c}=\mathrm{Softmax}\left(\mathrm{Cov}\left(F_{in},F_{e}\right)\right),$$

where $A_{c}\in R^{C\times C}$ represents attention weights derived from the covariance matrix, and the Softmax function ensures that the values in each row of the matrix sum up to 1, making them interpretable as attention scores. Specifically,

(8) $$\mathrm{Cov}\left(F_{in},F_{e}\right)=\frac{1}{HW}F_{in}^{\prime \prime }\cdot {\left(F_{e}^{\prime \prime }\right)}^{T}.$$

Meanwhile, another branch forms the attention map along pairwise positions from the two feature maps. The denoted $A_{p}\in R^{HW\times HW}$ is the spatial attention matrix derived from the covariance operation between the input feature map and the edge-enhanced feature map. It is designed to capture the relationships between spatial locations and highlight regions of interest, especially around the edges, which are critical for segmentation tasks such as polyp detection.

Covariance between the two feature maps for each pair of spatial locations is computed as follows:

(9) $$\mathrm{Cov}\left(F_{e},F_{in}\right)=\frac{1}{HW}{\left(F_{e}^{\prime \prime }\right)}^{T}\cdot F_{in}^{\prime \prime }.$$

Then,

(10) $$A_{p}=\mathrm{Softmax}\left(\mathrm{Cov}\left(F_{e},F_{in}\right)\right),$$

where $A_{p}\in R^{HW\times HW}$ stores the position-wise covariance attention weights.

Finally, $A_{c}$ and $A_{p}$ are utilized to refine the corresponding input feature map. Therefore, the output feature map, $F_{out}\in R^{C\times H\times W}$, is generated.

### 3.4. Pipeline of CSEE

**Figure 4:**
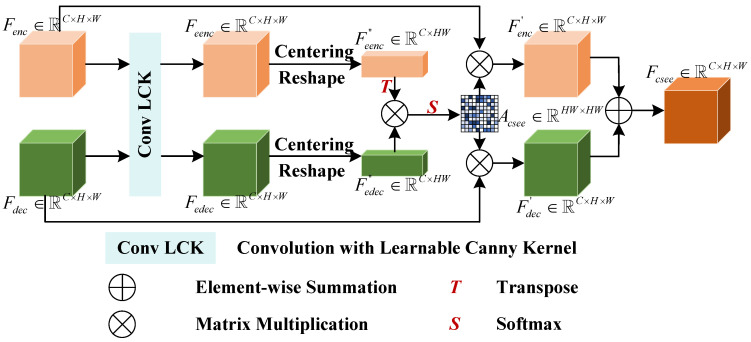
Pipeline of CSEE.

As shown in Figure 4, the proposed CSEE module is designed to bridge the gap between multi-scale feature maps in an encoder–decoder architecture. This module aims to preserve edge information across different resolutions and ensure consistency in boundary detection for segmentation tasks. In this section, we provide a detailed explanation of the CSEE pipeline, focusing on its multi-scale edge feature fusion and attention mechanisms. The CSEE module integrates edge information from both the encoder and decoder paths, ensuring that fine-grained boundary details are consistently represented across scales. It applies edge enhancement through a learnable Canny kernel and then computes spatial and channel-wise attention to align multi-scale features effectively.

Given the encoder feature map $F_{enc}\in R^{C\times H\times W}$ and decoder feature map $F_{dec}\in R^{C\times H\times W}$, we first extract edge feature using learnable Canny kernel convolution:

(11) $$F_{eenc}=\mathrm{ConvLCK}\left(F_{enc}\right),\;F_{edec}=\mathrm{ConvLCK}\left(F_{dec}\right).$$

Next, centring and reshaping operations are implemented:

(12) $${\bar{F}}_{eenc,c}=\frac{1}{HW}\sum_{i=1}^{HW}F_{eenc,c,i},\;{\bar{F}}_{edec,c}=\frac{1}{HW}\sum_{i=1}^{HW}F_{edec,c,i},$$

(13) $$F_{eenc}^{\prime }=F_{eenc}-{\bar{F}}_{eenc},\;F_{edec}^{\prime }=F_{edec}-{\bar{F}}_{edec}.$$

The reshaped feature maps are $F_{eenc}^{\prime \prime },F_{edec}^{\prime \prime }\in R^{C\times HW}$. Then, we deploy the covariance-based channel attention by carrying out the following:

(14) $$\mathrm{Cov}\left(F_{eenc},F_{edec}\right)=\frac{1}{HW}F_{eenc}^{\prime \prime }\cdot {\left(F_{edec}^{\prime \prime }\right)}^{T}.$$

Formally, the attention map can be expressed as follows:

(15) $$A_{csee}=\mathrm{Softmax}\left(\mathrm{Cov}\left(F_{eenc},F_{edec}\right)\right).$$

With the post-fusion by element-wise summation, the output feature map is obtained, $F_{csee}\in R^{C\times H\times W}$.

Overall, the CSEE module enhances polyp segmentation by ensuring that multi-scale edge information is retained and accurately fused. Key contributions of the CSEE module include the following:

1. The CSEE module aligns encoder and decoder feature maps across scales, ensuring that high-resolution and low-resolution features contribute equally to segmentation accuracy;
2. By using channel-wise attention, the CSEE module focuses on the most relevant channels, allowing the network to better capture the edge structures that are critical for precise segmentation;
3. The use of a learnable Canny kernel ensures that boundary information is consistently extracted and preserved, which is essential for distinguishing polyps from surrounding tissues in medical images.

### 3.5. Hybrid Loss Function

In this section, we propose a hybrid loss function designed to improve the accuracy of polyp segmentation by combining cross-entropy loss and edge-aware loss. The hybrid loss function is specifically tailored for the architecture integrating the CEEA and CSEE modules, which focus on both channel-wise and edge-preserving features. The goal of this loss function is to enhance not only the semantic segmentation accuracy but also the precision of boundary detection, which is critical for medical image segmentation tasks.

The hybrid loss function, denoted as $L_{\mathrm{hybrid}}$, is defined as a weighted sum of cross-entropy loss $L_{\mathrm{CE}}$ and edge-aware loss $L_{\mathrm{edge}}$:

(16) $$L_{\mathrm{hybrid}}=\alpha \cdot L_{\mathrm{CE}}+\beta \cdot L_{\mathrm{edge}},$$

where α and β are weights balancing the contributions of the two components (both set as 0.5 in this study). The cross-entropy loss measures the pixel-wise classification error. For a predicted segmentation map, $P\in R^{C\times H\times W}$, and ground truth, $Y\in R^{C\times H\times W}$, the cross-entropy loss is as follows:

(17) $$L_{\mathrm{CE}}=-\frac{1}{HW}\sum_{i=1}^{H}\sum_{j=1}^{W}\sum_{c=1}^{C}Y_{i,j,c}\log\left(P_{i,j,c}\right).$$

The edge-aware loss ensures accurate boundary detection by comparing the predicted and ground truth edge maps. First, edges are extracted:

(18) $$E_{\mathrm{GT}}=\mathrm{Edge}\left(Y\right),\;E_{P}=\mathrm{Edge}\left(P\right).$$

To sum up, this hybrid loss encourages both accurate segmentation and precise boundary detection, making it effective for polyp segmentation, where boundary precision is crucial.

## 4. Experiments

### 4.1. Datasets

Our experiments were conducted on two benchmark datasets, and the details are given in this subsection.

#### 4.1.1. Kvasir-SEG

As presented in Table 1, the Kvasir-SEG dataset, introduced by Jha et al. [32], is a large-scale dataset specifically designed for the task of polyp segmentation in colonoscopy images. It contains 1000 colonoscopy images with pixel-wise annotations for polyps, enabling researchers to evaluate the performance of various segmentation models. The images in the dataset cover a wide range of polyp sizes, shapes, and appearances, reflecting real-world variability in clinical colonoscopy procedures. The Kvasir-SEG dataset provides a robust benchmark for developing deep learning-based segmentation models, especially in the context of detecting and delineating polyps accurately. Additionally, the dataset is freely available, making it an important resource for both medical image analysis and broader multimedia modeling research.

#### 4.1.2. CVC-ClinicDB

As presented in Table 1, the CVC-ClinicDB dataset, introduced by Bernal et al. [33] in their work on WM-DOVA maps for polyp detection, is a widely used benchmark in colonoscopy image analysis. This dataset consists of 612 images extracted from colonoscopy video sequences, where each image is annotated with corresponding pixel-level ground truth masks of polyps. The dataset is designed to evaluate the performance of polyp detection and segmentation algorithms, providing a reliable benchmark for both traditional methods and deep learning-based approaches. The diversity in polyp appearance, size, shape, and texture makes CVC-ClinicDB a challenging dataset that closely reflects real clinical scenarios, which is crucial for developing robust medical image segmentation models. The dataset is validated against saliency maps provided by expert physicians, ensuring the accuracy and clinical relevance of the ground truth annotations.

### 4.2. Implement Details

The proposed EENet and the benchmark models were implemented on a Linux system, utilizing the PyTorch framework and accelerated by an NVIDIA A40 GPU. As presented in Table 2, to enhance model generalization, data augmentation methods, including random flipping and cropping, were applied. During training, we used a batch size of 64 with sub-patches sized at 256 × 256. The training procedure involved setting an initial learning rate of 0.02 and running for a maximum of 500 epochs. The optimization was handled by the SGD optimizer with momentum set at 0.9 and a polynomial learning rate decay strategy. The Softmax cross-entropy loss function was employed. The model achieving the lowest validation loss was selected for further evaluation.

We compared our EENet with several state-of-the-art methods, including UNet [47], DeepLav V3+ [14], UNet++ [15], ResUNet [49], ResUNet++ [16], PraNet [21], XNet [61], and Polyp-PVT [26].

### 4.3. Evaluation Metrics

In this study, we evaluated the performance of our predictions on the test set using four standard evaluation metrics:

(19) $$\mathrm{Dice}=\frac{2\times TP}{2\times TP+FP+FN},$$

(20) $$\mathrm{IoU}=\frac{TP}{TP+FP+FN},$$

(21) $$\mathrm{Sensitivity}=\frac{TP}{TP+FN},$$

(22) $$\mathrm{Specificity}=\frac{TN}{TN+FP},$$

where TP, TN, FP, and FN denote the counts of true positives, true negatives, false positives, and false negatives, respectively. Moreover, mDice and mIoU are calculated over all test sets.

### 4.4. Comparison with State-of-the-Art Models

#### 4.4.1. Numerical Evaluation of Kvasir-SEG

As presented in Table 3, the EENet exceeds all other models across all metrics. Notably, the EENet achieves a Dice coefficient of 0.9208, outperforming Polyp-PVT and PraNet, which scored 0.8907 and 0.8876, respectively. This Dice score improvement underscores EENet’s superior capability to accurately distinguish between polyp and non-polyp regions. Similarly, the EENet records the highest IoU of 0.8664, demonstrating its superiority in accurately delineating the boundaries of polyps, which is crucial in clinical settings.

In terms of sensitivity, the EENet achieved a score of 0.9912, indicating that the model is highly capable of identifying polyps across varying conditions, outperforming the next-best model, Polyp-PVT, which scored 0.9792. Lastly, the EENet also leads in specificity with a score of 0.9319, ensuring fewer false positives compared to other models, which is critical in reducing unnecessary follow-up procedures. These results validate the effectiveness of our CEEA and CSEE modules in enhancing polyp segmentation performance, particularly in challenging scenarios where precise boundary detection is crucial.

#### 4.4.2. Visual Inspections of Kvasir-SEG

**Figure 5:**
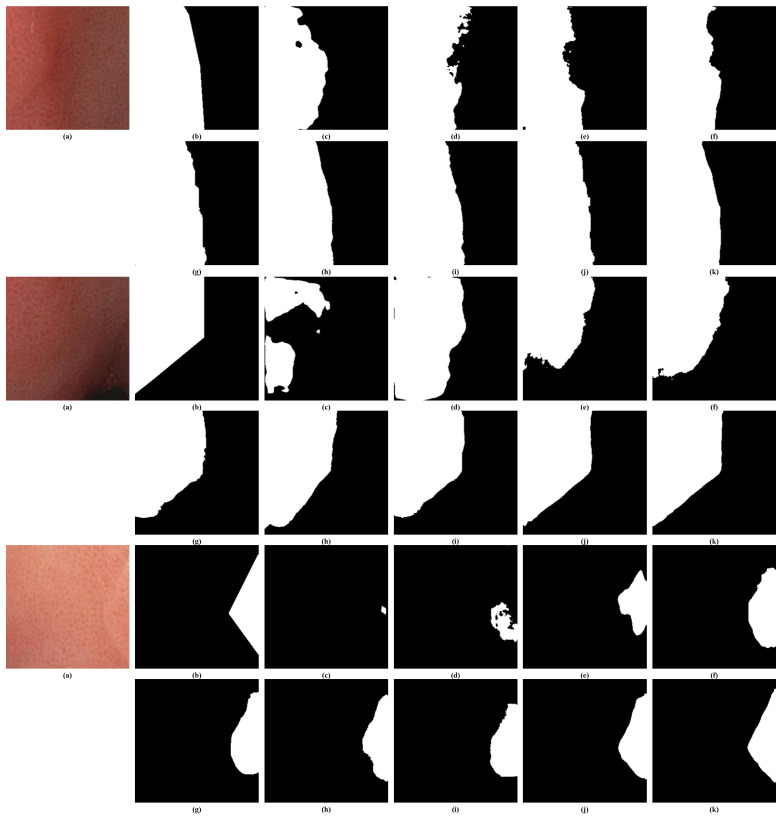
Visual inspections of random samples from Kvasir-SEG test set. (**a**) Input image, (**b**) ground truth, (**c**) UNet [47], (**d**) DeepLav V3+ [14], (**e**) UNet++ [15], (**f**) ResUNet [49], (**g**) ResUNet++ [16], (**h**) PraNet [21], (**i**) XNet [61], (**j**) Polyp-PVT [26], and (**k**) EENet (ours).

Figure 5 illustrates the qualitative comparisons of segmentation results on randomly selected samples from the Kvasir-SEG test set. From the visual inspections, we can observe that the EENet (k) consistently produces more precise and detailed polyp boundaries compared to the other models. Specifically, in regions where the polyp boundaries are irregular or faint, such as in the samples shown, the EENet is able to capture these subtle differences more accurately. Models such as Polyp-PVT (j) and PraNet (h) also perform well but tend to miss finer details, leading to slightly over-segmented or under-segmented areas. In contrast, traditional models like UNet (c) and UNet++ (e) show visible limitations, especially in handling complex polyp shapes and backgrounds, resulting in blurred or incomplete boundaries.

The effectiveness of the EENet is particularly evident in the cases where there is low contrast between the polyp and the surrounding tissues. It manages to segment the polyp with minimal false positives, and its results are visually closer to the ground truth compared to other models, indicating its robustness and superiority in real clinical scenarios.

#### 4.4.3. Numerical Evaluation of CVC-ClinicDB

Table 4 presents the quantitative results of our proposed EENet on the CVC-ClinicDB dataset, compared to several state-of-the-art models. The EENet achieves the best performance across most metrics. In terms of the Dice coefficient, the EENet records the highest score of 0.9316, outperforming Polyp-PVT (0.9178) and PraNet (0.8990). This reflects the EENet’s ability to segment polyps more accurately by capturing both fine details and overall structure. Furthermore, the EENet achieves the highest IoU score of 0.8817, indicating its effectiveness in delineating the boundaries of polyps with greater precision than other models, including Polyp-PVT (0.8667) and XNet (0.8204).

In terms of sensitivity, the EENet remains competitive with a score of 0.9915, closely matching the highest score of 0.9921 achieved by Polyp-PVT. This demonstrates that the EENet is highly capable of detecting polyps, including those with subtle or irregular boundaries. Importantly, the EENet shows a significant advantage in specificity, achieving the highest value of 0.9586, outperforming Polyp-PVT (0.9300) and PraNet (0.9110). The high specificity highlights the EENet’s ability to reduce false positives, ensuring that non-polyp regions are correctly identified, thus improving the overall robustness of the model in clinical applications.

Overall, these results demonstrate the superiority of the EENet in terms of both segmentation accuracy and boundary precision, validating the effectiveness of the CEEA and CSEE modules in handling complex polyp segmentation tasks.

#### 4.4.4. Visual Inspections of CVC-ClinicDB

**Figure 6:**
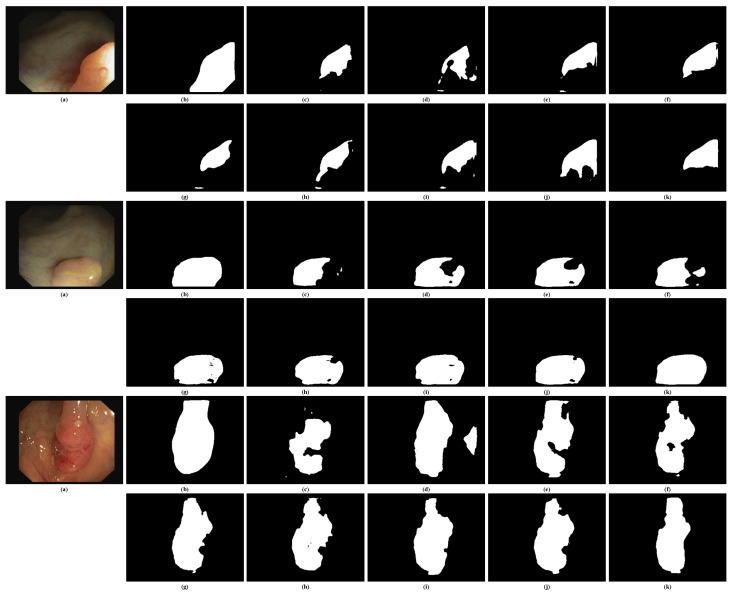
Visual inspections of random samples from CVC-ClinicDB test set. (**a**) Input image, (**b**) ground truth, (**c**) UNet [47], (**d**) DeepLav V3+ [14], (**e**) UNet++ [15], (**f**) ResUNet [49], (**g**) ResUNet++ [16], (**h**) PraNet [21], (**i**) XNet [61], (**j**) Polyp-PVT [26], and (**k**) EENet (ours).

Figure 6 provides visual comparisons of segmentation results from various models on randomly selected samples from the CVC-ClinicDB test set. Upon inspection, the EENet (k) demonstrates superior performance in capturing polyp boundaries compared to the other models. It consistently delivers the most accurate and sharp segmentation results, particularly in challenging regions where the polyp boundaries are less distinct or more complex. In contrast, models like UNet (c) and UNet++ (e) struggle with boundary precision, often producing over-segmented or under-segmented outputs, which can lead to incomplete or inaccurate delineation of polyps.

Advanced models such as Polyp-PVT (j) and PraNet (h) also perform well but occasionally miss finer details in the polyp structure, which can lead to slightly less accurate segmentation in comparison to the EENet. Overall, the EENet model stands out, providing segmentation results that are visually closer to the ground truth and exhibit greater precision, especially in the presence of challenging polyp structures and low-contrast regions. This further validates the effectiveness of the proposed network in real clinical scenarios where precise boundary detection is critical.

### 4.5. Ablation Study of CEEA

To evaluate the contribution of the CEEA module in the EENet, we performed an ablation study, replacing the CEEA layer with two alternatives: CB (see Figure 2) and a standard self-attention model [62]. The modified networks, referred to as EENet-C (with CB) and EENet-A (with self-attention), were tested on the Kvasir-SEG and CVC-ClinicDB datasets to assess the effect of the CEEA module on segmentation accuracy.

Table 5 presents the results of this ablation study. On the Kvasir-SEG dataset, EENet-C achieves a Dice/IoU score of 0.8687/0.8175, and EENet-A improves these results to 0.9023/0.8491, reflecting the benefit of introducing self-attention. However, when the full EENet with the CEEA module is used, the model reaches the best performance, achieving 0.9208/0.8664 on the same dataset. A similar trend is observed on the CVC-ClinicDB dataset, where EENet-C and EENet-A score 0.8508/0.8052 and 0.8977/0.8496, respectively, but the EENet with CEEA achieves the highest Dice/IoU of 0.9316/0.8817.

These results demonstrate the significant improvement brought upon by the CEEA module in both datasets. The incorporation of CEEA enhances both the accuracy and boundary precision of polyp segmentation, confirming the module’s effectiveness in capturing fine-grained details and spatial relationships that are crucial for high-quality segmentation performance.

### 4.6. Impacts of CSEE

To evaluate the contribution of the CSEE module in the overall performance of the EENet, we conducted experiments by removing the CSEE module from the network, resulting in the variant EENet without CSEE. The performance of this variant was then compared to that of the full EENet model on both the Kvasir-SEG and CVC-ClinicDB datasets.

Table 6 presents the results of this comparison. On the Kvasir-SEG dataset, the EENet without CSEE achieves a Dice/IoU score of 0.8795/0.8276, which shows a noticeable drop in performance compared to the full EENet, which scores 0.9208/0.8664. A similar pattern is observed in the CVC-ClinicDB dataset, where the EENet without CSEE achieves 0.8945/0.8465, while the full EENet reaches 0.9316/0.8817.

These results highlight the significant impact of the CSEE module on improving segmentation accuracy and boundary delineation. The CSEE module plays a crucial role in maintaining multi-scale consistency and enhancing boundary detection, leading to better segmentation outcomes, especially in challenging cases where precise edge detection is critical for accurate polyp segmentation. Removing CSEE results in a noticeable degradation in performance, reinforcing its importance in the overall network architecture.

### 4.7. Discussions

In this section, we provide a comprehensive analysis of the significance of our results, highlight the limitations of the EENet model, and suggest possible directions for further research. The experimental results demonstrate that the EENet achieves a Dice score of 0.9208 and an IoU of 0.8664 on the Kvasir-SEG dataset, outperforming models such as Polyp-PVT (0.8907 Dice, 0.8354 IoU) and PraNet (0.8876 Dice, 0.8303 IoU). Similarly, on the CVC-ClinicDB dataset, the EENet surpasses existing models with a Dice score of 0.9316 and an IoU of 0.8817, compared to Polyp-PVT (0.9178 Dice, 0.8667 IoU). These results represent a significant improvement in the accuracy and precision of polyp segmentation, especially in the detection of polyp boundaries. This higher accuracy is particularly crucial in clinical applications, where precise boundary delineation can reduce the risk of complications during polyp removal procedures.

However, the trade-off between accuracy and computational cost must be considered. While the EENet achieves superior segmentation results, the inclusion of the CEEA and CSEE modules increases the model’s complexity, potentially leading to longer processing times compared to simpler architectures like U-Net or ResUNet. This trade-off may affect the model’s real-time performance in clinical settings, where rapid image processing is critical. While the EENet has shown excellent performance on polyp segmentation datasets, its effectiveness on other medical segmentation tasks (e.g., tumor detection in different organs) has not yet been explored. The model may require fine-tuning or architectural adjustments to generalize effectively across other medical imaging modalities.

Specifically, while the EENet demonstrates excellent performance, with Dice scores above 92% on both the Kvasir-SEG and CVC-ClinicDB datasets, we recognize that for clinical diagnostic tools, accuracy must be as close to perfect as possible. Current models, including ours, serve as valuable second opinions in diagnostics, but for them to become primary tools, further improvements are necessary. Future work will focus on increasing accuracy through methods such as integrating multi-modal data, leveraging transformer-based architectures, and conducting extensive clinical validation. These efforts aim to ensure that models like the EENet can meet the stringent accuracy requirements of clinical practice, providing more reliable and safer diagnostic support.

## 5. Conclusions

This paper presents the edge-enhanced network (EENet), designed to improve the accuracy and boundary precision of polyp segmentation in colonoscopy images. The EENet achieved a Dice score of 0.9208 and an IoU of 0.8664 on the Kvasir-SEG dataset, and a Dice score of 0.9316 with an IoU of 0.8817 on the CVC-ClinicDB dataset, outperforming several state-of-the-art models such as Polyp-PVT and PraNet. Clinically, these improvements translate into more accurate and reliable polyp detection during colonoscopy procedures. The higher Dice and IoU scores suggest that the EENet can reduce false positives and false negatives, which is critical for preventing colorectal cancer by ensuring that polyps are accurately identified and removed. Improved boundary precision also means that less healthy tissue may be affected during polyp removal, minimizing the risk of complications. Therefore, the EENet not only provides technical improvements in segmentation performance but also offers significant potential for enhancing patient outcomes in real clinical settings.

In future work, we aim to extend the capabilities of the EENet by exploring its generalizability to other medical imaging tasks, such as segmentation of tumors and lesions in various organs beyond the colon. Additionally, incorporating advanced attention mechanisms, such as transformer-based architectures, could further enhance the model’s ability to capture long-range dependencies in complex medical images. Another avenue of research is to optimize the network for real-time performance, making it suitable for integration into clinical workflows where fast and accurate feedback is critical. Furthermore, we plan to explore the application of semi-supervised or unsupervised learning techniques to reduce the dependency on large labeled datasets, which are often challenging to obtain in medical contexts. These developments have the potential to further improve the robustness and applicability of the EENet in a variety of clinical settings.

## Figures and Tables

**Table 1:** Dataset properties.

Dataset	Size	Total	Training Set	Validation Set	Test Set
Kvasir-SEG [32]	487 × 332 to 1920 × 1072	1000	600	200	200
CVC-ClinicDB [33]	288 × 368	612	368	122	122

**Table 2:** Experimental settings.

Items	Settings
Learning strategy	Poly decay
Initial learning rate	0.002
Loss function	Cross-entropy
Max epoch	500
GPU memory	48 GB
Optimizer	SGD
Sub-patch size	256×256
Batch size	64

**Table 3:** Results on the Kvasir-SEG dataset, where the bold text indicates the best results.

Methods	Dice	IoU	Sensitivity	Specificity
UNet [47]	0.8120	0.7405	0.9430	0.8507
DeepLab V3+ [14]	0.8149	0.7432	0.9464	0.8538
UNet++ [15]	0.8109	0.7349	0.9739	0.7971
ResUNet [49]	0.8179	0.7459	0.9499	0.8569
ResUNet++ [16]	0.8245	0.7734	0.8937	0.8299
PraNet [21]	0.8876	0.8303	0.9667	0.9015
XNet [61]	0.8583	0.8076	0.9239	0.8686
Polyp-PVT [26]	0.8907	0.8354	0.9792	0.9088
EENet (ours)	**0.9208**	**0.8664**	**0.9912**	**0.9319**

**Table 4:** Results on the CVC-ClinicDB dataset, where the bold text indicates the best results.

Methods	Dice	IoU	Sensitivity	Specificity
UNet [47]	0.7618	0.6988	0.8766	0.7729
DeepLab V3+ [14]	0.7984	0.7325	0.9187	0.8101
UNet++ [15]	0.7940	0.7290	0.9270	0.7950
ResUNet [49]	0.7957	0.7299	0.9155	0.8073
ResUNet++ [16]	0.8590	0.7881	0.9885	0.8716
PraNet [21]	0.8990	0.8490	0.9901	0.9110
XNet [61]	0.8943	0.8204	0.9910	0.9073
Polyp-PVT [26]	0.9178	0.8667	**0.9921**	0.9300
EENet (ours)	**0.9316**	**0.8817**	0.9915	**0.9586**

**Table 5:** Results of different variants on two dataset.

Models	Kvasir-SEG	CVC-ClinicDB
EENet-C	0.8687/0.8175	0.8508/0.8052
EENet-A	0.9023/0.8491	0.8977/0.8496
EENet	0.9208/0.8664	0.9316/0.8817

**Table 6:** Results of removing CSEE.

Models	Kvasir-SEG	CVC-ClinicDB
EENet w/o CSEE	0.8795/0.8276	0.8945/0.8465
EENet	0.9208/0.8664	0.9316/0.8817

## Data Availability

Public datasets were used in this paper. The download links are [https://datasets.simula.no/kvasir-seg/], accessed on 10 December 2022, and [https://polyp.grand-challenge.org/CVCClinicDB/], accessed on 5 October 2021.
